# The Effects of Bazedoxifene on Bone, Glucose, and Lipid Metabolism in Postmenopausal Women With Type 2 Diabetes: An Exploratory Pilot Study

**DOI:** 10.14740/jocmr2278w

**Published:** 2015-08-23

**Authors:** Taishi Yoshii, Masayo Yamada, Taichi Minami, Tetsuji Tsunoda, Mayuko Sasaki, Yoshinobu Kondo, Shinobu Satoh, Yasuo Terauchi

**Affiliations:** a Division of Metabolism and Endocrinology, Department of Internal Medicine, Yokohama Sakae Kyosai Hospital, Federation of National Public Service Personnel Mutual Associations Yokohama, 132 Katsura-cho, Sakae-ku, Yokohama, Kanagawa 247-8581, Japan; b Department of Endocrinology and Metabolism, Graduate School of Medicine, Yokohama City University, 3-9 Fukuura, Kanazawa-ku, Yokohama, Kanagawa 236-0004, Japan; c Department of Endocrinology and Metabolism, Chigasaki Municipal Hospital, 5-15-1 Motomura, Chigasaki, Kanagawa 253-0042, Japan

**Keywords:** Bazedoxifene, Type 2 diabetes mellitus, Bone metabolism, Selective estrogen receptor modulators, Postmenopausal women

## Abstract

**Background:**

Selective estrogen receptor modulators (SERMs) decrease homocysteine and cross-linking of pentosidine and reduce low-density lipoprotein cholesterol (LDL-C), and they are expected to improve bone quality and atherosclerosis. Therefore, the potential effects of bazedoxifene on bone (bone resorption, bone formation, and bone quality), as well as on glucose and lipid metabolism markers, were examined in Japanese postmenopausal women with type 2 diabetes mellitus (T2DM).

**Methods:**

Eligible patients received 20 mg of bazedoxifene tablets once daily and were followed up for 12 weeks. Bone resorption markers including tartrate-resistant acid phosphatase 5b (TRACP-5b), bone formation markers and bone quality markers such as homocysteine and serum pentosidine, total cholesterol (TC), LDL-C, high-density lipoprotein cholesterol (HDL-C), triglycerides (TG), and HbA1c were all measured.

**Results:**

Twenty patients completed this study. All bone resorption markers decreased significantly 4 weeks after bazedoxifene treatment. In particular, TRACP-5b decreased significantly at 12 weeks (median percent change: -20.6%), and the minimum significant change (MSC) achievement rate of TRACP-5b was 65%. Bazedoxifene also decreased bone formation markers. However, bazedoxifene did not improve bone quality markers. LDL-C, HDL-C, and non-HDL-C were decreased, but TG was unchanged. Glucose metabolism was not changed after bazedoxifene treatment. In a subgroup analysis, the group of patients in whom the percent change in TRACP-5b exceeded the MSC had no change in pentosidine levels at 12 weeks. However, in the group of patients in whom the percent change in TRACP-5b did not exceed the MSC, pentosidine levels tended to increase.

**Conclusions:**

Bazedoxifene may improve bone resorption markers and LDL-C without affecting glucose metabolism in Japanese postmenopausal women with T2DM.

## Introduction

Persistent hyperglycemia in patients with diabetes mellitus is said to be associated with various complications. Bone fragility is one of these diabetic complications that have attracted attention. Insulin deficiency impairs osteoblast formation and function, and as a result, insulin deficiency is associated with decreased bone mass [1, 2]. Therefore, bone mineral density (BMD) in patients with type 1 diabetes mellitus (T1DM) is lower than in those with type 2 diabetes mellitus (T2DM). However, despite BMD not being low in patients with T2DM, a higher risk of fractures has been reported [3].

Bone strength is determined by BMD and bone quality, and the high risk of fracture in T2DM is thought to be due to reduced bone quality [4]. Increased homocysteine and pentosidine accumulation, advanced glycation end products (AGEs), leads to an increase in non-enzymatic cross-linking in bone, thus reducing bone quality [5-7]. The mechanism by which homocysteine and pentosidine increase in diabetes is thought to be as follows. First, excessive gluconeogenesis due to hyperglycemia causes a deficiency in vitamin B6. As a result, this vitamin B6 deficiency leads to higher homocysteine levels [8, 9]. Then, increased glycation and increased oxidative stress due to persistent hyperglycemia promote the formation of pentosidine cross-linking in bone [10].

Selective estrogen receptor modulators (SERMs) have estrogen-like effects that reduce bone resorption and increase BMD. SERMs have also been reported to have antioxidant effects [11]. These antioxidant effects of SERMs may decrease homocysteine levels [12, 13]. In addition, in a rabbit model of reduced bone quality, the antioxidant effects of SERMs decreased pentosidine accumulation [14]. Thus, SERMs are promising drugs to improve bone quality. SERMs may also be beneficial in patients with diabetes mellitus with increased homocysteine and pentosidine accumulation. Moreover, SERMs do not affect glucose metabolism [15], but their specific estrogen-like effect improves lipid metabolism [16]. Therefore, SERMs may also be effective in reducing the progression of arteriosclerosis in patients with diabetes mellitus.

Bazedoxifene is a novel third-generation SERM for the treatment of postmenopausal osteoporosis. Bazedoxifene, compared to raloxifene, reduced the risk of non-vertebral fractures in a high-risk fracture group [17]. Therefore, bazedoxifene may be an effective option for treatment of postmenopausal osteoporosis. However, to the best of our knowledge, the effects of bazedoxifene on bone, glucose, and lipid metabolism in postmenopausal patients with T2DM have not been reported.

The primary purpose of this pilot study was to explore the potential effects of bazedoxifene on bone metabolism in Japanese postmenopausal patients with T2DM. In addition, the effects of bazedoxifene on glucose and lipid metabolism markers and on indices of arteriosclerosis were also investigated.

## Materials and Methods

### Study protocol/patients

This pilot study was performed as a multicenter, prospective, observational study between February 2013 and September 2013 at three institutes in Japan. This study was approved by the ethics committees of each participating site and it was performed in accordance with the Declaration of Helsinki. This study was registered with the UMIN Clinical Trial Registry (UMIN 000010074).

Patients with T2DM who regularly visited the hospital were assessed for eligibility for this study. Inclusion criteria were as follows: 1) > 2 years since menopause; 2) < 85 years of age; 3) no fluctuations in HbA1c levels > 0.5% in the past 3 months; 4) HbA1c levels < 8.4% over the past 3 months; 5) body mass index (BMI) < 30 kg/m^2^; 6) estimated glomerular filtration rate (eGFR) levels > 30 mL/min/1.73 m^2^; 7) no requirement for new or changed diabetes drug regimens within 4 weeks of starting bazedoxifene treatment; and 8) no requirement for new or changed angiotensin II receptor antagonist, statin, or proton pump inhibitor regimens within 4 weeks of starting bazedoxifene treatment.

Exclusion criteria were as follows: 1) previous history of non-traumatic bone fracture; 2) thiazolidine medication; 3) steroid hormone medication; 4) oral medication for abnormal thyroid function; 5) other medications, including active vitamin D_3_ drugs, vitamin K_2_, raloxifene, bisphosphonate, denosumab, or parathyroid hormone (PTH) treatment for osteoporosis; 6) history of deep vein thrombosis; or 7) the presence of any malignancies.

In the present study, the baseline BMD value was not included in the eligibility criteria because T2DM patients are at high risk for fracture due to reduced bone quality despite BMD not being low. Therefore, patients were recruited regardless of the baseline BMD value. All patients provided their written informed consent prior to participation in the study.

Eligible patients received 20 mg of bazedoxifene tablets once daily and were followed up for 12 weeks. During the 12-week study period, no changes or additions to drugs for treatment of diabetes mellitus, hypertension, or lipid metabolism were permitted. In addition, no additional drugs for treatment of osteoporosis were allowed.

Primary endpoints were changes in the bone metabolism markers, including bone resorption markers (urinary type I collagen cross-linked N-telopeptide (u-NTX), urinary type I collagen cross-linked C-telopeptide (u-CTX), and tartrate-resistant acid phosphatase 5b (TRACP-5b)), bone formation markers (osteocalcin (OC) and undercarboxylated osteocalcin (ucOC)), and bone quality markers (homocysteine (HC) and serum pentosidine (PS)). Secondary endpoints were changes in HbA1c, high molecular weight (HMW) adiponectin, and the lipid metabolism markers (total cholesterol (TC), low-density lipoprotein cholesterol (LDL-C), high-density lipoprotein cholesterol (HDL-C), and triglycerides (TG)).

### Assessments

To evaluate the patients’ condition at baseline (week 0), lumbar spine and femoral neck bone mineral densities were assessed using T-scores and young adult mean (YAM) values from dual-energy X-ray absorptiometry (Discovery X, HOLOGIC), and the fracture risk assessment tool (FRAX^®^) created by the World Health Organization was used as an index of the risk for major osteoporotic and hip fractures within the next 10 years.

Overnight fasting blood and urine samples were collected, and weight was measured at 0, 4, and 12 weeks after bazedoxifene treatment.

To evaluate bone metabolism, the bone resorption, formation, and quality markers were measured at the central research center of SRL (SRL, Inc., Tokyo, Japan).

The minimum significant change (MSC) of the bone resorption markers was used for measuring the effect of bazedoxifene. The MSC is used for measuring the effects of osteoporosis therapies. The MSC of u-NTX, u-CTX, and TRACP-5b has been defined as 27.3%, 23.5%, and 12.4%, respectively [18, 19]. When the percent changes of bone resorption markers after treatments were over the MSC, the osteoporosis therapy was considered effective.

To evaluate glucose metabolism, fasting plasma glucose (FPG) (by the glucose oxidation method, chemical reagent and Glucose AUTO, and STAT GA-1160 analyzer; Arkray Inc., Kyoto, Japan), fasting insulin (SRL, Inc.), fasting plasma C-peptide (SRL, Inc.), HbA1c levels (Adams A1c HA-8160, Arkray Inc.), and HMW adiponectin (SRL, Inc.) were measured at 0, 4, and 12 weeks. HOMA-IR and HOMA-β represent insulin resistance and pancreatic β-cell function, respectively, and were calculated as follows: HOMA-IR = fasting insulin (μU/mL) × fasting plasma glucose (mg/dL)/22.5, and HOMA-β = 20 × fasting insulin (μU/mL)/(fasting plasma glucose (mg/dL) - 3.5).

To evaluate lipid metabolism, TC, LDL-C, HDL-C, and TG (Kyowa Medex, Tokyo, Japan) were measured at 0, 4, and 12 weeks. Non-HDL-C (mg/dL) was calculated as follows: TC (mg/dL) - HDL-C (mg/dL).

The levels of other markers, aspartate amino transaminase (AST), alanine amino transaminase (ALT), gamma-glutamyl transpeptidase (γGTP), and serum creatinine (Cr), were measured at 0, 4, and 12 weeks. The urinary albumin-to-creatinine ratio (UACR) and cystatin C levels were measured at 0 and 12 weeks. Brachial ankle pulse wave velocity (baPWV) (VaSera VS-1000, Fukuda Denshi), high sensitivity C-reactive protein (hs-CRP), and ferritin levels were measured as arteriosclerosis indices at 0 and 12 weeks.

### Adverse events

Safety was assessed on the basis of all reported adverse events identified by the physicians’ examinations and blood parameters.

### Statistical analysis

Statistical analysis was conducted using the Statistical Package for the Social Sciences (SPSS version 11.0.1 J, SPSS Inc., Armonk, NY).

Baseline characteristics are summarized descriptively. To investigate the effects of the intervention, the median value was calculated at each point, and the change from baseline was tested using the Wilcoxon signed-rank test at each post-treatment point. Median percent change from baseline was also calculated. In addition, the MSC achievement rates were computed as the proportion of patients who achieved an improvement exceeding the MSC for each bone resorption marker. Moreover, subgroup analyses were conducted to explore the effects of bazedoxifene on bone, glucose, and lipid metabolism markers based on glycemic control at baseline and whether they exceeded the MSC of TRACP-5b at 12 weeks. For the analysis of HOMA-IR and HOMA-β, patients on insulin therapy were excluded.

The level of statistical significance was set at 0.05. Due to the exploratory nature of the study, no correction for multiple significance testing was made.

## Results

Of the 23 patients enrolled, 20 completed this study. Two patients withdrew from this trial before starting the administration of bazedoxifene; one was found to be ineligible after enrollment, and the other withdrew consent. One patient was withdrawn after the administration of bazedoxifene because of suspected angina pectoris.

The patients’ baseline characteristics are shown in Table 1. The mean age (± standard deviation) was 67.9 ± 5.5 years, and the mean BMI was 22.5 ± 2.8 kg/m^2^. These patients were not obese. All patients had good glucose control (median HbA1c 6.3%) and short diabetes duration (median 4.5 years) in the present study. Femoral neck BMD was moderately decreased, but lumbar BMD was normal. All patients had no clinical and morphometric fractures.

**Table 1:** Patients’ Baseline Characteristics

	Baseline (n = 20)
Age (years)	67.9 ± 5.5
Menopause age (years)	50.2 ± 5.1
Diabetes duration (years)	4.5 (1 - 24)
Height (cm)	153.5 ± 4.4
BMI (kg/m^2^)	22.5 ± 2.8
HbA1c (%)	6.3 (5.4 - 7.4)
Serum creatinine (mg/dL)	0.61 (0.39 - 0.82)
Microangiopathy	
Neuropathy (%)	29 (5/17)
Retinopathy (%)	0 (0/17)
Nephropathy (%)	
Stage 1	82 (14/17)
Stage 2	18 (3/17)
Macroangiopathy	
Coronary artery disease (%)	5.9 (1/17)
Cerebrovascular disease (%)	24 (4/17)
Peripheral artery disease (%)	0 (0/17)
Smoking (%)	
Former smoker	20 (5/20)
Current smoker	0 (0/20)
Alcohol (%)	0 (0/20)
Lumbar	
Bone mineral density (g/cm^2^)	0.9 ± 0.2
T-score	-1.3 ± 1.6
YAM (%)	86.4 ± 17.0
Femoral neck	
Bone mineral density (g/cm^2^)	0.6 ± 0.1
T-score	-1.6 ± 0.8
YAM (%)	78.2 ± 10.9
FRAX^®^ tool	
Major osteoporotic (years)	8.3 (5.2 - 24)
Hip fracture (years)	1.3 (0.4 - 7.6)
History of the vertebral fracture	0 (0/20)
History of the non-vertebral fracture	0 (0/20)
Family history of the femoral neck fracture	0 (0/20)
Medications	
Anti-diabetes	
Diet only (%)	15 (3/20)
Sulfonylureas (%)	20 (4/20)
Glinides (%)	10 (2/20)
Biguanides (%)	25 (5/20)
DPP-IV inhibitors (%)	45 (9/20)
GLP-1 analogues (%)	10 (2/20)
α-glucosidase inhibitors (%)	5 (1/20)
Insulin (%)	5 (1/20)
Anti-dyslipidemia	
Stains (%)	35 (7/20)
Ezetimibe (%)	15 (3/20)
Anti-hypertension	
ARBs (%)	30 (6/20)

Data are reported as means ± standard deviation or medians (min - max). BMI: body mass index; YAM: young adult mean; FRAX^®^ tool: Fracture Risk Assessment Tool; ARBs: angiotensin II receptor blocker.

All clinical parameters, such as bone, glucose, lipid, and other levels, are shown in Table 2.

**Table 2:** Values of All Clinical Parameters From 0 to 12 Weeks

	0 weeks	4 weeks	12 weeks	P value vs. 4 weeks	P value vs. 12 weeks
uNTX (nmol BCE/mmol CRE)	36.9 (16.4 - 126)	28.3 (15.2 - 66.9)	29.7 (20.5 - 74.4)	0.008	NS
uCTX (μg/mmol CRE)	232 (57 - 666)	180 (57-390)	181 (70-414)	0.011	NS
TRACP-5b (mU/dL)	325 (155 - 606)	257 (149 - 472)	259 (141 - 388)	< 0.001	< 0.001
Osteocalcin (ng/mL)	7.7 (4.6 - 13)	7.8 (5.3 - 12)	7.1 (5.3 - 10)	NS	NS
Undercarboxylated osteocalcin (ng/mL)	3.6 (0.9 - 12)	3.7 (1.6 - 10)	3.3 (1.6 - 6.9)	0.002	0.04
Homocysteine (mmol/mL)	6.1 (4.5 - 11.7)	6.2 (4.6 - 13.2)	6.4 (4.3 - 12.4)	NS	NS
Serum pentosidine (μg/mL)	0.042 (0.022 - 0.065)	0.043 (0.031 - 0.069)	0.045 (0.038 - 0.076)	NS	NS
Fasting plasma glucose (mg/dL)	112 (75 - 174)	100 (88 - 158)	105 (78 - 161)	NS	NS
HbA1c (%)	6.3 (5.4 - 7.4)	6.4 (5.5 - 7.3)	6.2 (5.5 - 7.0)	NS	NS
IRI (μU/mL)	5.31 (2.21 - 10.7)	5.33 (2.24 - 11.6)	5.54 (2.28 - 12.8)	NS	NS
sCPR (ng/mL)	1.42 (0.33 - 2.73)	1.4 (0.88 - 3.09)	1.47 (0.14 - 2.86)	NS	NS
HOMA-IR**	1.4 (0.5 - 3.2)	1.3 (0.5 - 2.8)	1.5 (0.9 - 3.4)	0.02	NS
HOMA-β**	45.5 (10.3 - 155)	41.7 (8.49 - 139)	45.4 (8.83 - 107)	NS	NS
HMW adiponectin (μg/mL)	3.46 (1.32 - 10.9)	-	3.37 (1.42 - 12.8)	-	0.024
LDL cholesterol (mg/dL)	124 (53.0 - 154)	107 (58.0 - 151)	108 (50.0 - 168)	0.003	0.037
HDL cholesterol (mg/dL)	76.5 (37.0 - 1 06)	72 (45.0 - 108)	71 (43.0 - 108)	NS	0.027
Non-HDL cholesterol (mg/dL)	130 (64.0 - 167)	117 (70.0 - 157)	115 (58.0 - 170)	0.001	0.01
Triglyceride (mg/dL)	86.5 (44.0 - 258)	108 (42.0 - 187)	110 (39.0 - 193)	NS	NS
baPWV (cm/s)					
Rt	1,884 (1,243 - 2,545)	-	1,808 (1,201 - 2,972)	-	NS
Lt	1,867 (1,241 - 2,687)	-	1,836 (1,201 - 3,032)	-	NS
hs-CRP (ng/mL)	431 (86 - 4,520)	563 (148 - 7,120)	468 (109 - 4,990)	NS	NS
Ferritin (ng/mL)	96 (7.1 - 256)	-	90.8 (5.1 - 299)	-	NS
AST (IU/L)	21 (13 - 43)	21 (11 - 42)	20 (12 - 63)	NS	NS
ALT (IU/L)	19 (9 - 48)	17 (7 - 55)	19 (7 - 64)	NS	NS
γ-GTP(IU/L)	19 (12 - 45)	18 (11 - 47)	19 (13 - 47)	NS	NS
Serum Cr (mg/dL)	0.61 (0.39 - 0.82)	0.61 (0.43 - 0.81)	0.61 (0.39 - 0.81)	NS	NS
UACR (mg/g CRE)	13.5 (3.7 - 42.8)	-	10.1 (2.1 - 80.4)	-	NS
Cystatin C (mg/L)	0.59 (0.41 - 0.84)	-	0.55 (0.33 - 0.87)	-	0.009
Weight (kg)	54.5 (42.5 - 70)	54.6 (42.2 - 69)	55 (42.3 - 70)	NS	NS

*Data are reported as medians (min - max). **Patients with insulin therapy (n = 1) were excluded from analysis of HOMA-IR and HOMA-β. UACR: urinary albumin-to-creatinine ratio; NS: no significance.

### Bone resorption markers

The levels of u-NTX, u-CTX, and TRACP-5b decreased significantly from baseline after 4 weeks of bazedoxifene treatment (P = 0.008, P = 0.011, and P < 0.001, respectively). In particular, TRACP-5b levels decreased significantly at 12 weeks.

**Figure 1:**
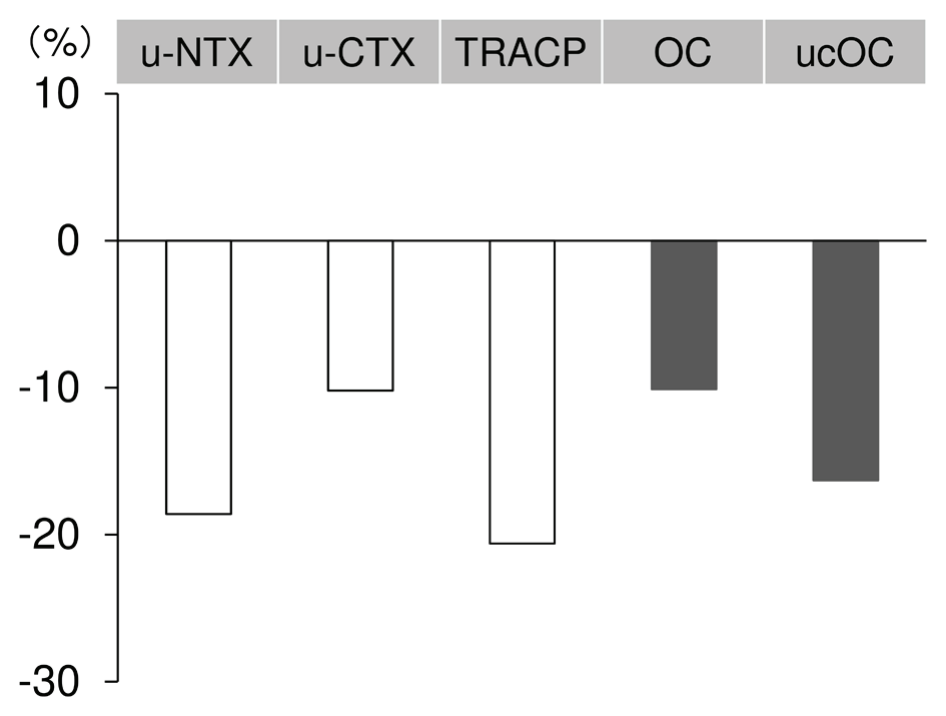
Median percent changes in bone metabolism after 12 weeks of bazedoxifene treatment. Median values (first quartile, third quartile) are -18.6% (-30.9%, 12.9%) for u-NTX, -10.2% (-44.1%, 20.7%) for u-CTX, -20.6% (-27.8%, -5.8%) for TRACP, -10.1% (-17.7%, 8.6%) for OC, and -16.3% (-23.4%, -2.2%) for ucOC. u-NTX: urinary type I collagen cross-linked N-telopeptide; u-CTX: urinary type I collagen cross-linked C-telopeptide; TRACP: tartrate-resistant acid phosphatase 5b; OC: osteocalcin; ucOC: undercarboxylated osteocalcin.

Median percent changes in bone resorption and formation makers after 12 weeks of treatment with bazedoxifene are shown in Figure 1. The median percent changes of u-NTX, u-CTX, and TRACP-5b at 12 weeks were -18.6%, -10.2%, and -20.6%, respectively (Fig. 1). The median percent change exceeded the MSC only for TRACP-5b.

The MSC achievement rate at 12 weeks was the highest for TRACP-5b (65%), followed by u-CTX (45%) and u-NTX (30%).

### Bone formation markers

The OC levels remained stable over the 12 weeks, but the ucOC levels were significantly decreased at 12 weeks (P = 0.040).

### Bone quality markers

Homocysteine and serum pentosidine levels were not changed after administration of bazedoxifene.

### Lipid metabolism

Bazedoxifene significantly reduced LDL-C levels from 124 (53.0 - 154) (median, (min - max)) mg/dL at baseline to 107 (58.0 - 151) mg/dL and 108 (50.0 - 168) mg/dL at 4 and 12 weeks (P = 0.003, P = 0.037), respectively. The LDL-C median percent changes from baseline were -11.6% and -6.2% at 4 and 12 weeks, respectively. HDL-C levels and non-HDL-C levels decreased from 76.5 (37.0 - 106) mg/dL and 130 (64.0 - 167) mg/dL at baseline to 71.0 (43.0 - 108) mg/dL and 115 (58.0 - 170) mg/dL at 12 weeks (P = 0.027, P = 0.010), respectively. TG levels were unchanged.

### Glucose metabolism

HbA1c, FPG, HOMA-IR, and HOMA-β were relatively stable over the 12 weeks. However, HMW adiponectin levels decreased significantly at 12 weeks (P = 0.024).

### Other markers

The indices of atherosclerosis, baPWV (Rt/Lt), hs-CRP, and ferritin were unchanged. In addition, AST, ALT, and γ-GTP levels did not change after bazedoxifene treatment. Serum creatinine levels were also stable, but cystatin C levels were significantly decreased at 12 weeks.

### Adverse events

During the study period, no serious adverse events were reported. One suspected case of angina pectoris was observed using a Masters two-step test, but no stenosis was seen on coronary angiography. Other adverse events reportedly related to bazedoxifene treatment such as thrombosis, hepatic dysfunction, or hot flushes were not observed.

### Subgroup analyses

In a subgroup analysis of the effects of bazedoxifene based on glycemic control at baseline, the median percent changes in TRACP-5b at 12 weeks were -18.0% in the HbA1c < 6.5% group (n = 11) and -25.3% in the HbA1c ≥ 6.5% group (n = 9). Both groups did not have the statistical significant difference.

Additionally, in a subgroup analysis based on the TRACP-5b reduction effect of bazedoxifene, the pentosidine levels were stable in the group of patients (n = 13) in whom the percent change in TRACP-5b exceeded the MSC (from 0.043 μg/mL at baseline to 0.044 μg/mL at 12 weeks). However, the pentosidine level tended to increase in the group of patients (n = 7) in whom the percent change in TRACP-5b did not exceed the MSC (from 0.034 μg/mL at baseline to 0.050 μg/mL at 12 weeks).

## Discussion

The present study is the first to investigate the effects of bazedoxifene in postmenopausal patients with T2DM in an exploratory manner. In this study of Japanese postmenopausal patients with T2DM, bazedoxifene improved bone turnover and reduced LDL-C without affecting glucose metabolism.

After starting bazedoxifene, all bone resorption markers (u-NTX, u-CTX, and TRACP-5b) decreased after 4 weeks and remained low from week 4 to week 12; the changes from baseline to 4 weeks were significant. In particular, TRACP-5b decreased significantly after 12 weeks of treatment with bazedoxifene. TRACP-5b is a serum bone resorption marker reflecting osteoclast activity. TRACP-5b, compared to other bone resorption markers, has less diurnal variation, and it is not affected by renal function or diet [20]. Therefore, TRACP-5b is thought to be useful to diagnose osteoporosis and assess the effectiveness of treatment. The MSC for percent improvement in TRACP-5b is reported to be 12.4% [19].

In the present study, the percent improvement in TRACP-5b following treatment with bazedoxifene was 20.6%, with an MSC achievement rate of 65%, thus showing that bazedoxifene improved bone resorption in patients with T2DM, as well. In a subgroup analysis of the effects of bazedoxifene based on glycemic control before treatment, the median percent changes in TRACP-5b at 12 weeks of treatment with bazedoxifene were -18.0% in the HbA1c < 6.5% group and -25.3% in the HbA1c ≥ 6.5% group. This might suggest that bazedoxifene is effective in improving bone resorption in diabetic patients with poor glycemic control, but the mechanism leading to the observed change remains unclear, and further examinations are needed.

Bazedoxifene decreased bone formation markers in this study. These results are similar to previously reported results. This is thought to be because these bone formation markers, such as OC and ucOC, decreased secondary to the reduction in bone resorption by bazedoxifene.

Bazedoxifene did not decrease bone quality markers such as homocysteine or serum pentosidine in this study. High levels of homocysteine and pentosidine have been reported to be clinical risk factors for fractures. In the Rotterdam study, the relative risk of all fractures with 1-SD higher log homocysteine levels increased by 1.4 (95% confidence interval 1.2 - 1.6) [21]. High urinary pentosidine levels were an independent predictive factor for new fracture development in postmenopausal women [22]. Serum pentosidine levels are also associated with existing fractures, independent of age and BMD, in postmenopausal patients with T2DM [6].

With regard to the bone quality improvement effects of SERMs, raloxifene has been reported to decrease homocysteine levels and to decrease pentosidine cross-linking [12, 14]. However, no consensus has yet been reached on the mechanism of improvement. Moreover, improvement of serum and urinary pentosidine levels with SERMs has not been clinically reported [23]. Therefore, whether there were any differences in the changes of pentosidine levels associated with the TRACP-5b reduction effects of bazedoxifene was also examined. With treatment with bazedoxifene, the group of patients in whom the percent change in TRACP-5b exceeded the MSC had no change in pentosidine levels at 12 weeks. However, in the group of patients in whom the percent change in TRACP-5b did not exceed the MSC, pentosidine levels increased. These findings suggest that greater TRACP-5b reduction effects of bazedoxifene may inhibit an increase in pentosidine levels.

In the present study, the reason for the stable bone quality markers was probably that homocysteine and serum pentosidine levels at baseline were lower in the study patients than in previous reports. The median homocysteine concentration in the present study was 6.1 nmol/L, which is not hyperhomocysteinemia (cut-off level 15 nmol/L). In addition, AGEs such as pentosidine are known to be higher in diabetic patients with poor glycemic control and longer diabetes duration [24]. Because the present study patients had relatively good glycemic control and a shorter duration of diabetes, there was probably not enough accumulation of AGEs. Therefore, it was thought that baseline levels of homocysteine and serum pentosidine were too low to observe the improvement with bazedoxifene.

With respect to lipid metabolism, raloxifene and bazedoxifene have previously been reported to decrease LDL-C, but no consensus has been achieved about the effects on HDL-C or TG [25, 26]. HDL-C is reported to increase with raloxifene and bazedoxifene [12, 17]. In the present study, bazedoxifene decreased both LDL-C and HDL-C levels. However, bazedoxifene also decreased non-HDL-C levels. Therefore, we believe that bazedoxifene improves lipid metabolism. Whether the LDL-C lowering and HDL-C increasing effects of SERMs also have an anti-arteriosclerotic effect has not been clearly answered. Raloxifene improves flow-mediated vasodilatation (FMD) and intima-media thickness (IMT) [27, 28]. Raloxifene also decreases inflammatory cytokines such as TNF-α, IL-6, and MCP-1 that cause arteriosclerosis [29-31]. In large-scale clinical studies, however, raloxifene has not been reported to reduce cardiovascular or cerebrovascular events [16, 32]. In the present study, arteriosclerosis indices such as hs-CRP and baPWV were not improved.

With respect to glucose metabolism, as previously reported with raloxifene [15], treatment with bazedoxifene in the present study produced no changes in glycemic control, insulin resistance, and insulin secretion in humans, and raloxifene has the report to suggest possibility to prevent the onset of diabetes in rats [33]. However, bazedoxifene did decrease adiponectin levels. Adiponectin is mainly affected by visceral fat mass and ucOC [34, 35]. In particular, adiponectin decreases with an increase in visceral fat mass. The present study patients had no weight changes, so the decrease in adiponectin may have been associated with a decrease in the bone formation marker ucOC. In OC knockout mice, adiponectin is decreased, whereas in Esp-/- mice, which have high OC levels with opposite characteristics, the ucOC concentration compared to total OC is high, and adiponectin is increased. This suggests that ucOC may regulate adiponectin. It is suggested that SERMs secondarily decrease ucOC, and this may lead to a decrease in adiponectin.

The present study has a few limitations. First, this study had a small sample size and no control group. However, this study was a pilot study, and no information was available to calculate the sample size. The second limitation was the short study period. A period of 12 weeks was set in a manner similar to many studies of the evaluation of bone resorption markers [26]. However, it might be short in terms of the evaluation of the changes in bone formation and quality markers. Re-evaluation of bone formation markers after the administration of SERMs is recommended 24 weeks later [18], and a study reported that bone quality markers improved 24 weeks after the administration of SERMs [23]. Further studies may be needed with a study period longer than 12 weeks.

Bazedoxifene tended to be more effective in preventing bone resorption in the HbA1c ≥ 6.5% group than in the HbA1c < 6.5% group. This suggests that the bone resorption improvement effects of bazedoxifene may differ depending on the degree of baseline glycemic control. Therefore, further studies on the effectiveness of bazedoxifene in T2DM patients with poor glycemic control should be conducted. In addition, in the bazedoxifene-responsive group based on TRACP-5b reduction, elevation of pentosidine tended to be prevented. Thus, pentosidine levels may be useful in assessing the bone quality improvement effects of bazedoxifene, and further studies in a larger number of patients would be desirable.

The present study, which was exploratory in nature, may be very valuable for constructing hypotheses leading to future studies and for establishing new treatments. However, no causal relationship between treatment with bazedoxifene and effectiveness can be demonstrated by the present study results alone. Large-scale comparative studies are needed for more confirmatory evidence [36, 37].

In conclusion, bazedoxifene may improve bone resorption markers and LDL-C levels without affecting glucose metabolism in postmenopausal patients with T2DM with good glycemic control.
